# The Acute and Delayed Mortality of the Northern Krill (*Meganyctiphanes norvegica*) When Exposed to Hydrogen Peroxide

**DOI:** 10.1007/s00128-020-02996-6

**Published:** 2020-09-26

**Authors:** Rosa H. Escobar-Lux, Ole B. Samuelsen

**Affiliations:** 1 Institute of Marine Research, Austevoll Research Station, Sauganeset 16, 5392 Storebø, Norway; 2 Institute of Marine Research, Nordnes, P.O. Box 1870, 5817 Bergen, Norway

**Keywords:** Crustacean, Toxicity, LC_50_, Aquaculture

## Abstract

Bath treatment pharmaceuticals used to control sea lice infestations in the salmonid industry, such as hydrogen peroxide (H_2_O_2_), are released directly into the environment where non-target organisms are at risk of exposure. The aim of this study was to determine the threshold concentrations for mortality of the Northern krill, *Meganyctiphanes norvegica,* a major component of the north Atlantic marine ecosystem. To assess the lethal effects of H_2_O_2_, we carried out a series of 1 h acute toxicity tests and assessed mortality through a 48 h post-exposure period. One-hour exposure to 170 mg/L, corresponding to 10% of the recommended H_2_O_2_ treatment, caused 100% mortality and a subsequent acute median-lethal concentration LC50 value of 32.5 mg/L. Increased mortality was observed with time in all exposed groups, resulting in successively lower LC_50_ values during the post-exposure period. The suggested H_2_O_2_ concentrations have the potential of causing negative effects to the Northern krill.

Sea lice (*Lepeophtheirus salmonis* and *Caligus rogercresseyi*), naturally occurring parasitic copepods affecting both farmed and wild salmonid populations, are a major challenge for the salmonid industry worldwide (Costello 2006; Torrissen et al. 2013; Vollset et al. 2016). The parasites feed on the mucous, skin, and blood of its host, and if present in significant numbers they can cause damage associated with osmotic stress and secondary infections (Finstad et al. 2000; Johnson et al. 2004; González et al. 2015). Norwegian wild salmonid populations, migrating post smolts from Atlantic salmon and local populations of sea trout (*Salmon trutta*), can suffer high mortality if there is high density of salmon lice larvae in the surrounding water (Costello 2009; Vollset et al. 2016). In farmed fish, salmon lice infestations reduce the general welfare of the fish and lead to an increase of the overall cost of the industry due to reduced growth and marketability due to skin lesions, and high costs associated with delousing treatments (Costello 2009). Therefore, both the economic and ecological impact of salmon lice infestations are significant challenges for the salmonid industry.

In order to control salmon lice infestations, the industry has relied on the use of different chemotherapeutants, through the application of bath treatments and the use of in-feed drugs. Bath treatments can be applied either by enclosing the fish cages with an impervious tarpaulin or transferring the fish into well-boats, and after treatment the waste water is directly released into the surrounding water (Ernst et al. 2001; Burridge et al. 2010). At a global level, hydrogen peroxide (H_2_O_2_) was introduced as an antiparasitic agent after the loss of sensitivity in both *L. salmonis* and *C. rogercresseyi* to other delousing agents (Bravo et al. 2015; Urbina et al. 2019). In Norway alone, H_2_O_2_ is still the most used bath treatment therapeutant with a consumption of 4523 tons in 2019 (www.fhi.no/hn/legemiddelbruk).

Hydrogen peroxide acts on salmon lice by hydroxyl radicals attacking lipid and cellular organelles resulting in inactivation of enzymes and DNA replication (Cotran et al. 1989; Urbina et al. 2019). Previous studies have also shown that decomposition of hydrogen peroxide to water and O_2_ bubbles in the gut and the haemolymph may cause mechanical paralysis leading to detachment of the pre-adult and adult salmon lice from the fish and causing them to float towards the surface (Bruno and Raynard 1994; Aaen et al. 2014). A bath treatment involves the release of a large volume of H_2_O_2_ containing waste water and the chemical can potentially be dispersed over a wide area (Burridge et al. 2010, 2014, Parsons et al. 2020, Refseth et al. 2017). Therefore, there is a growing concern about the possible toxic effects of H_2_O_2_ on non-target aquatic invertebrate species living in the vicinity of fish farms, and specifically crustaceans which has been proven as particularly vulnerable (Smit et al. 2008; Burridge et al. 2014; Van Geest et al. 2014; Gebauer et al. 2017; Hansen et al. 2017; Bechmann et al. 2019; Escobar-Lux et al. 2019).

The pelagic zooplankton, *Meganyctiphanes norvegica,* Northern krill, is a species at risk as its distribution overlaps with the location to many salmon farms in Norway, as it inhabits both coastal and offshore waters (Kaartvedt et al. 2002; Melle et al. 2004; Tarling et al. 2010). Furthermore, the distribution of this boreal krill species has been described to be seasonal, with a predominant coastal distribution between the months of January and May (Grover 1952). In Norway, during this period of the year, pharmaceuticals are being used to keep the level of salmon lice below 0.2 female lice per fish as specified in the Norwegian Ministry of Trade, Industry and Fisheries (FOR-2012-12-05-1140, 2012) (Grefsrud et al. 2019). The total biomass of euphasiid stocks in the Norwegian Sea has been previously estimated to 42 million tons (Mt), with around 40–75% of this stock being Northern krill (Lindley 1982; Melle et al. 2004). Thus, the northern krill is a major component of the north Atlantic marine ecosystem, acting as a keystone organism between lower trophic levels and larger predators and plays an important role in the sequestration of carbon (Kaartvedt et al. 2005; Tarling et al. 2010). It is preyed upon by several commercially important fish species (Sameoto et al. 1994; Onsrud et al. 2004), seabirds (Montevecchi et al. 1992; Stevick et al. 2008), and marine mammals (Brodie et al. 1978). Moreover, the commercial exploitation of Northern krill is gaining interest in the salmonid industry as a potential protein alternative to the fishmeal (Tarling et al. 2010). Mass death of krill washed up on a beach can occur and is considered a natural phenomenon. Previously the mass stranding of *M. norvegica* has been explained as predation events in which predators’ chase krill ashore (MacDonald 1927), transported to land by oceanic currents or by special events like upwellings (Aitken 1960; Cox 1975), or because special lightning conditions that might interfere with the krill’s behavior (Wiborg 1966). However, in recent years there has been a higher frequency of reports in Norway describing this phenomenon near areas with salmon farms. This started a debate in public media of what might have caused the mass mortality and one of the most frequently cited suggestions has been the use of pesticides for delousing of the salmon farms, and especially H_2_O_2_. However, the effects of H_2_O_2_ exposure on the Northern krill have until now been unknown.

For treating salmon, the recommended concentration for a H_2_O_2_ bath treatment is 1500–2100 mg/L for 20 min depending on temperature (https://www.felleskatalogen.no/medisin-vet). Typically, toxicity studies use exposure times that vary from 24 to 96 h. However, these may not be representative of the real-life scenarios following a release of waste water after a bath treatment on a salmon farm (Ernst et al. 2001; Urbina et al. 2019). The use of 1 h exposures, is considered a more realistic exposure scenario, but to date only a limited number of species have been tested under those conditions (Medina et al. 2004; Fairchild et al. 2010; Burridge et al. 2014; Van Geest et al. 2014; Escobar-Lux et al. 2019; Parsons et al. 2020). What these previous studies also have shown is that the mortality observed immediately after exposure tends to be lower than the mortalities registered if a post-exposure period is included in the experimental set-up. A longer post-exposure observation period is therefore recommended.

The main objective of this study was to examine the toxicity of H_2_O_2_ to *M. norvegica*, a non-target crustacean and keystone species of the Norwegian marine environment. Our objective was to expose the Northern krill to a short 1 h pulse of H_2_O_2_ and assess the acute and delayed mortality during a post-exposure period of 48 h in clean seawater.

## Materials and Methods

In the present study, krill (*M. norvegica*) were collected from the dock at Austevoll Research Station, Institute of Marine Research Norway (60° 05′ 20″ N 5° 15′ 57″ E) using light traps. The light traps (mesh size 500 µm; 0.45 m in diameter; BellaMare USA) were equipped with a white LED light and deployed at a depth of 20 m overnight. The research station is at least 3 km away from the nearest commercial salmon farm. Krill from the traps were transported to the laboratory at Austevoll Research Station and kept overnight in 10 L buckets supplied with sand filtered seawater from a depth of 160 m (Bjørnafjorden) holding a temperature of 8 °C (salinity of 34.2 ppt; pH 7.94). The experiment was performed within 48 h of capture and prior to exposure the krill were sorted and only krill in excellent physical condition were used in the experiments.

Commercial H_2_O_2_ (Nemona, Akzo Nobel Pulp and Performance Chemicals AB Sweden) at a concentration of 49.50% (600 g/L) was purchased from Akzo Nobel, Pulp and Performance Chemicals, AB Sweden. Since no previous studies had assessed the toxicity of H_2_O_2_ on *M. norvegica*, the chosen concentrations were based on the recommended dose for treating salmon (1700 mg/L). The krill were exposed to concentrations of 1.7, 8.5, 17, 170, 850 and 1700 mg/L H_2_O_2_, corresponding to 0.1, 0.5, 1, 10, 50 and 100% of the recommended treatment dose. All exposures were conducted in glassware units with a volume of 500 mL. A total of 140 krill were randomly divided into seven treatment groups, including a control group, with four replicates for each treatment and each replicate counting five individuals. After the 1 h exposure, acute mortality was recorded and the krill were transferred to 10 L recovery tanks where mortality was checked successively at 6, 24 and 48 h post-exposure using a dissecting microscope. Krill were considered dead if there was no movement of the pereopods, pleopods or antenna after a gentle stimulus. Mortality that occurred during the 1 h exposure was defined as acute mortality. Total mortality was defined as the cumulative mortality after the 48 h post-exposure period.

The statistical analyses for mortality were done in the software R (Version 3.5.3 (2019-03-11) Copyright © 2019 The R Foundation for Statistical Computing). The LC_50_ values, and their 95% confidence intervals (CI), were calculated using generalized linear models (GLM) with binomial error structures and probit links, according to Finney (1971). Hydrogen peroxide concentrations were log10 transformed to linearize the data.

## Results and Discussion

**Fig. 1:**
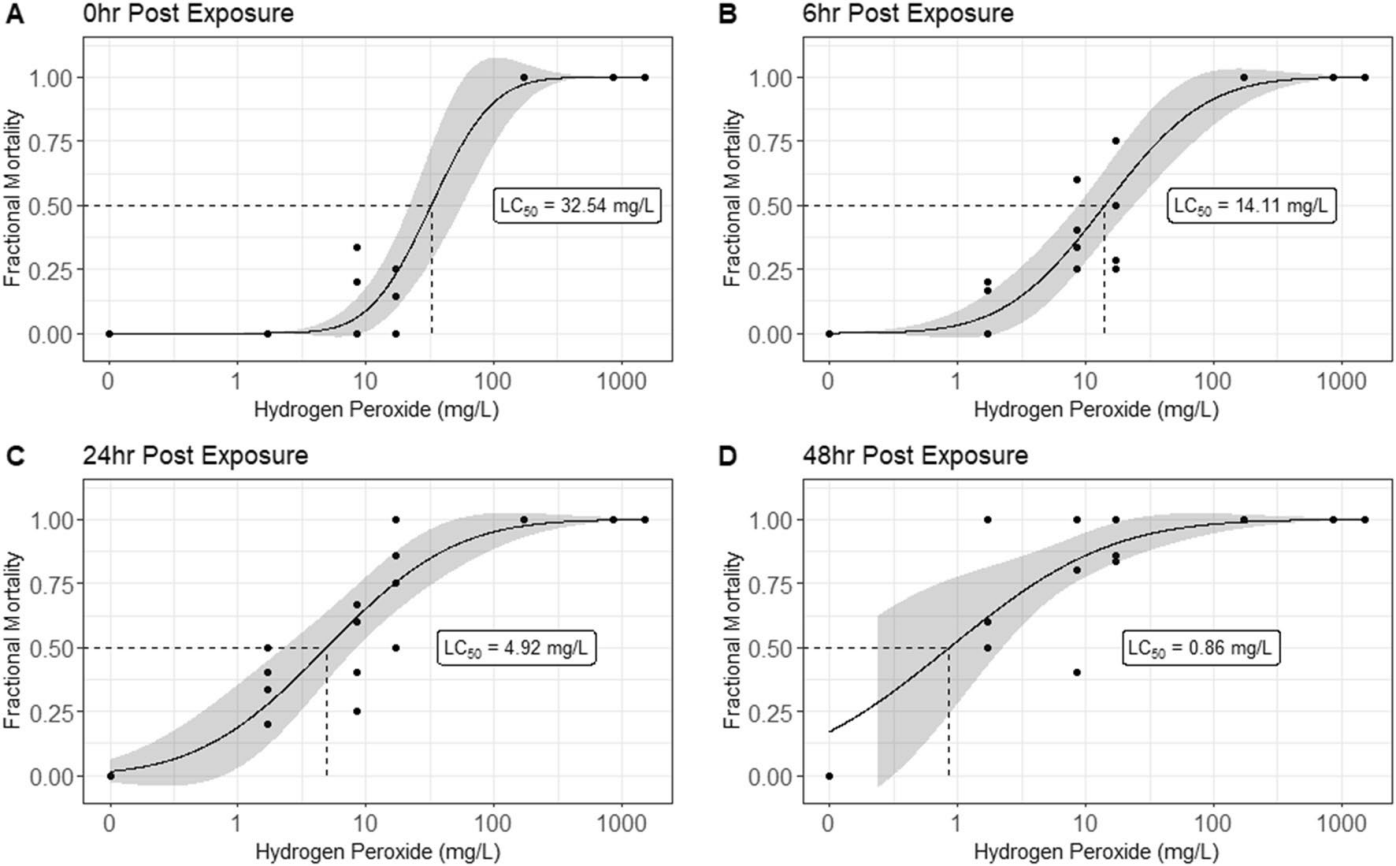
The toxicity of hydrogen peroxide to *M. norvegica* following 1 h exposure. Dose–response curves showing mortality amongst the northern krill at 0 h, 6 h, 24 h, and 48 h post-exposure to H_2_O_2._ Each point on the graphs represent an individual replicate tank containing 4 to 6 krill and the line represent the best fit model for the data calculated using a binomial log-probit GLM in R. The shadowed area represents the 95% confidence intervals

This study clearly show that H_2_O_2_ was acutely toxic to wild-captured Northern krill *M. norvegica*. While no mortality was recorded in the group exposed to the lowest dose of 1.7 mg/L or in the control group, a 1 h exposure to 170 mg/L, i.e. 10% of recommended dose, caused 100% mortality and a subsequent acute LC_50_ value of 32.5 mg/L (16.8–48.2) was calculated (Fig. 1a). During the post-exposure period, increased mortality with time was observed in all exposed groups resulting in successively lower LC_50_ values with 14.11 mg/L after 6 h (7.3–20.9), 4.92 mg/L (1.2–7.9) after 24 h and finally 0.86 mg/L after 48 h (Fig. 1b–d). No mortality was registered in the control groups during the post-exposure period. The calculated LC_50_ value at 24 h represents a threefold dilution of the acute 1 h LC_50_ value. These findings clearly support the recommendations suggested in previous studies to include a post-exposure period following the exposure to H_2_O_2_ to assess any delayed effects (Van Geest et al. 2014; Brokke 2015; Escobar-Lux et al. 2019).

While several studies have examined the toxicity of H_2_O_2_ on marine crustacean species, the number of studies using an exposure time of 1 h is more limited. A review of those studies reveals that some crustaceans have a relatively high tolerance to H_2_O_2_ exposure and is reflected in low mortality when exposed to concentrations similar to or higher than the recommended treatment dose. This applies to both newly hatched larvae and adult of American lobster (*Homarus americanus*), sand shrimp (*Crangon septemspinosa*), the mysid *Mysid* sp. (Burridge et al. 2014), rock pool shrimp (*Palaemon elegans*) and chameleon shrimp (*Praunus flexuosus*) (Brokke 2015). For some species, low mortality was observed even when a post-exposure period was included in the study. Following an exposure of 1 h and a 95 h post-exposure period, the calculated LC_50_ values were 1673 mg/L for *H. americanus* larvae, > 3750 mg/L for adult American lobster, 3182 mg/L for sand shrimps and 973 mg/L for *Mysid* sp. (Burridge et al. 2014; Van Geest et al. 2014). For rock pool shrimps and chameleon shrimps the acute mortality after 1 h exposure was low indicating LC_50_ values higher than the highest exposure concentration of 1700 mg/L for both species (Brokke 2015). However, a significant mortality occurred during the 24 h post-exposure period, resulting in LC_50_ values of 174.1 mg/L and 77.5 mg/L for rock pool shrimp and chameleon shrimps respectively, classifying these species as highly sensitive. In the study by Bechmann et al. (2019), the Northern shrimp (*Pandalus borealis*) was exposed to 15 mg/L H_2_O_2_ for 1 h. The very low acute mortality observed immediately after exposure did however increase during the post-exposure period (7 days) but as the total mortality never exceeded 30%, no LC_50_ could be calculated. Damage on the gills was observed in the shrimps exposed to H_2_O_2_ and suggested as the major cause of the delayed mortality (Bechmann et al. 2019).

In comparison, species like the copepods *Acartia Hudsonica* and *Calanus* spp. have shown higher sensitivity to H_2_O_2_ exposure, resulting in EC_50_ and LC_50_ values of 2.6–10 mg/L and 30.6 mg/L respectively, following a 24 h post-exposure period (Van Geest et al. 2014; Escobar-Lux et al. 2019). In the case of the European lobster (*Homarus gammarus*) larvae (stage I–IV), a 1 h exposure to 1530 mg/L followed by a 24 h post-exposure period, resulted in mortalities between 75 and 100% (Escobar-Lux et al. 2020) and calculated LC_50_ values of 177 mg/L, 404 mg/L, 676 mg/L and 738 mg/L, for stages I, II, III and IV respectively. For species other than crustaceans, the polychaete *Ophryotrocha* sp. and the sugar kelp *Saccharina latissima* are amongst the more sensitive marine species with LD_50_ values of 64.3 mg/L and 80.7 mg/L following 72 h and 7 days’ post-exposure periods, respectively (Fang et al. 2018; Haugland et al. 2019). The LC_50_ values calculated for northern krill are therefore, to our knowledge the most sensitive species examined so far.

This study has shown that a bath treatment with H_2_O_2_ has a detrimental effect on *M. norvegica.* However, it is important to assess whether these laboratory-based concentrations are likely to pose a significant risk to krill at the proximity of salmonid aquaculture sites. Due to differences in experimental set-ups the variation in half-lives reported for H_2_O_2_ in seawater in large, with results between 1 and 58 days (Bruno and Raynard 1994; Lyons et al. 2014; Fagereng 2016; Parsons and Samuelsen unpubl. data). Several factors affect both the toxicity and the degradation of H_2_O_2_, for example the water temperature or the irradiance (Stratford et al. 1984; Treasure et al. 2000)”. However, even the shortest degradation time reported (1 day) is significantly longer than the 1 h exposure needed in the present study to cause considerable mortality of the Northern krill. Even though H_2_O_2_ is extensively used around the world as an anti-sea lice bath treatment, few studies have initiated the use of mathematical models to predict its’ dispersal and its’ impact on non-target species. One such study from Norway has indicated that the spread of H_2_O_2_ may be larger than previously thought (Refseth et al. 2017). According to the model, concentrations up to 300 mg/L may occur within a 1 km radius from the farm and 100 mg/L within a radius of 2 km. Furthermore, the model also suggested that a concentration of 100 mg/L can be present in surface waters for several hours after discharge. The presented model simulations therefore suggest that the Northern krill within 2 km of a salmonid farm may be exposed to lethal concentration of H_2_O_2_.

Parsons et al. (2020) used dispersion models to predict the spreading of pharmaceuticals from salmonid farms in Norway, following bath treatment. Based on the models and LC_50_ values (1 h exposure followed by 24 h post-exposure period) for European lobster larvae (stage I and II) they calculated impact zones around 23 Norwegian fish farms for the pesticides azamethiphos and deltamethrin. This model however, did not take into account the degradation of the compounds due to the presence of organic matter in the water. While the azamethiphos impact zones around farms were relatively small (mean area of 0.04–0.2 km^2^), deltamethrin impact zones covered much larger areas (mean area of 21.1–39.0 km^2^). The difference in impact zone is due to the difference in toxicity between the two drugs. For azamethiphos the 1 h-LC_50_ values (95% CIs) for stage I and II larvae were 43.1 μg/L (13.0–131.0 μg/L) and 20.5 μg/L (13.2–30.9 mg/L), respectively, representing approximately 2- and fivefold dilutions of the treatment concentration (100 μg/L) used on Norwegian fish farms. For deltamethrin the 1 h-LC_50_ values (with 95% CIs) for stage I and II larvae were estimated to be 2.6 ng/L (0.6–11.0 ng/L) and 2.9 ng/L (1.5–5.7 ng/L), representing approximately 800-fold dilution of the treatment concentration of 2000 ng/L. Considering the sensitivity of krill towards H_2_O_2_ found in the present study, where the LC_50_ ranged from 52- to 2000-fold dilution with increasing post-exposure period, impact zones like those calculated for deltamethrin in Parsons et al. (2020) will be most relevant for impact zones for H_2_O_2_ and krill.

*Meganyctiphanes norvegica* can be found around the North Atlantic, with the Norwegian sea being a major hotspot for its distribution (Melle et al. 2004). Due to their distribution, krill can often be found in waters close to aquaculture sites and therefore be negatively impacted by the dispersal of effluent plumes after treatments. Based on our findings and the information from previous mathematical models, H_2_O_2_ may cause a larger impact than it was previously believed. Therefore, that some cases of mass mortality of krill observed in past years may have been caused by H_2_O_2_ exposure, cannot be overlooked.
